# Redefining QRS transition to confirm left bundle branch capture during left bundle branch area pacing

**DOI:** 10.3389/fcvm.2023.1217133

**Published:** 2023-07-14

**Authors:** Sem Briongos-Figuero, Álvaro Estévez Paniagua, Ana Sánchez Hernández, Roberto Muñoz-Aguilera

**Affiliations:** Cardiology Department, Infanta Leonor Hospital, Madrid, Spain

**Keywords:** QRS transition, left bundle branch capture, left ventricular septal capture, lead screwing-in, diagnostic performance

## Abstract

**Background:**

QRS transition criteria during dynamic manoeuvers are the gold-standard for non-invasive confirmation of left bundle branch (LBB) capture, but they are seen in <50% of LBB area pacing (LBBAP) procedures.

**Objective:**

We hypothesized that transition from left ventricular septal pacing (LVSP) to LBB pacing (LBBP), when observed during lead penetration into the deep interventricular septum (IVS) with interrupted pacemapping, can suggest LBB capture.

**Methods:**

QRS transition during lead screwing-in was defined as shortening of paced V6-R wave peak time (RWPT) by ≥10 ms from LVSP to non-selective LBBP (ns-LBBP) obtained during mid to deep septal lead progression at the same target area, between two consecutive pacing manoeuvres. ECG-based criteria were used to compared LVSP and ns-LBBP morphologies obtained by interrupted pacemapping.

**Results:**

Sixty patients with demonstrated transition from LVSP to ns-LBBP during dynamic manoeuvers were compared to 44 patients with the same transition during lead screwing-in. Average shortening in paced V6-RWPT was similar among study groups (17.3 ± 6.8 ms vs. 18.8 ± 4.9 ms for transition during dynamic manoeuvres and lead screwing-in, respectively; *p* = 0.719). Paced V6-RWPT and aVL-RWPT, V6-V1 interpeak interval and the recently described LBBP score, were also similar for ns-LBBP morphologies in both groups. LVSP morphologies showed longer V6-RWPT and aVL-RWPT, shorter V6-V1 interpeak interval and lower LBBP score punctuation, without differences among the two QRS transition groups. V6-RWPT < 75 ms or V6-V1 interpeak interval > 44 ms criterion was more frequently achieved in ns-LBBP morphologies obtained during lead screwing-in compared to those obtained during dynamic manoeuvres (70.5% vs. 50%, respectively *p* = 0.036).

**Conclusions:**

During LBBAP procedure, QRS transition from LVSP to ns-LBBP can be observed as the lead penetrates deep into the IVS with interrupted pacemapping. Shortening of at least 10 ms in paced V6-RWPT may serve as marker of LBB capture.

## 1. Introduction

Left bundle branch (LBB) area pacing can provide physiological pacing through two different types of capture: left ventricular septal myocardial pacing (LVSP) or direct LBB capture via non-selective (ns)-LBB or selective (s)-LBB pacing. Both LBB area pacing (LBBAP) modalities [LVSP and LBB pacing (LBBP)] have demonstrated shorter QRS duration, improved electro-mechanical left ventricular (LV) synchrony, and better clinical outcomes when compared to conventional right ventricular pacing among bradycardia patients (1, 2). However, direct capture of the LBB provides faster LV activation than LVSP, generates superior electrical and mechanical resynchronization (3), and it results in better LV ejection fraction improvement and higher rates of clinical and echocardiographic response among heart failure patients with broad QRS complex (4).

Nowadays, the discrimination between ns-LBB capture and deep left septal myocardial capture remains one of the challenges of LBBAP (5). The electrophysiological demonstration of early retrograde His potential or early distal anterograde left conduction system potential during LBBAP offers 100% specificity for the confirmation of LBB capture, but relies on invasive procedures which are difficult to generalize in clinical practice (6). Thus, QRS morphology transition criteria demonstrated during dynamic electrocardiographic (ECG) manoeuvres (differential output pacing or programmed stimulation) are the current gold-standard for the non-invasive confirmation of LBB capture. An abrupt prolongation of the paced R wave peak time (RWPT) in lead V6 corroborates the loss of LBB capture (6, 7). However, sensitivity of the dynamic ECG manoeuvres to discriminate LBB capture is usually low and in many cases it is not possible to demonstrate differential captures because of the close proximity in pacing thresholds of the cardiac conduction system and the adjacent myocardium (8).

We hypothesize that transition from LVSP morphology to ns-LBBP morphology, when observed during the lead screwing into the deep interventricular septum with interrupted pacemapping, can also suggest direct LBB capture during LBBAP implantation.

## 2. Material and methods

### 2.1. Study design

This study enrolled all consecutive patients with an attempt of LBBAP procedure for bradycardia and/or heart failure indications (as bailed-out strategy) from February 2020 to March 2023 at our institution. Baseline clinical data and procedure-related data were acquired in a prospective way.

We defined QRS transition from LVSP to ns-LBBP capture during interrupted lead screwing-in pacemapping. Then, we compared LVSP and ns-LBBP morphologies obtained in cases in which transition was demonstrated during dynamic ECG manoeuvres using differential output pacing, with LVSP and ns-LBBP morphologies obtained in cases in which transition was observed during lead screwing-in. ECG-based criteria (paced V6-RWPT and V6-V1 interpeak interval) (9, 10) were used for the comparison, along with the paced aVL-RWPT and LBBP score, recently described by our group (11). Finally, we analysed the accomplishment of currently accepted 100% specific cut-off values (12), V6-RWPT < 75 ms or V6-V1 interpeak interval > 44 ms, as surrogates of LBB capture.

The study adhered to the Helsinki Declaration as revised in 2013 and the Institutional Bioethical Committee approved the research protocol. All patients were informed about the nature of the conduction system pacing device and provided informed consent.

### 2.2. Procedure description

In our laboratory, LBBAP implantation is routinely performed using the “single lead technique” (13–15). All procedures were performed using the fix curve Medtronic C315His sheath and the 4.1F, active helix, screw-in pacing lead (model 3830; Medtronic). First, the most superior aspect of the tricuspid annulus, appraised fluoroscopically, was used as anatomical landmark. We targeted the area located approximately 2 cm apically from the tricuspid annulus, extending conically 2 cm to the superior and inferior midseptum, to perform right ventricular septal pacemapping before the lead deployment. Ideal paced QRS morphologies were those with QS and notched nadir in V1 with discordant polarity in lead II and III (R wave in lead II with RS/rS/QS morphology in lead III). The lead screwing-in process was then initiated by rapid rotations. Interrupted pacing from the lead was provided to monitor changes in the paced QRS morphology during the penetration process. The paced QRS morphology was considered optimal if significant QRS narrowing occurred, along with the disappearance of the LBB block pattern and the appearance of QR/Qr or rsR′/rSr′ pattern in lead V1 and RS or R morphology in lead V5/V6. At this point, QRS measurements and differential output pacing were performed to confirm LBB capture. We did not perform deep septal stimulation. If the optimal paced QRS morphology was not obtained or deep septal lead penetration was not possible, the lead was extracted and other areas of the interventricular septum were targeted, such as those with paced QRS morphologies suggesting superior locations (R wave in lead II and III) or inferior locations (rS/QS in lead II and III). We did not routinely use contrast injection through the delivery sheath.

Procedures were recorded on a digital electrophysiological system (General Electric, USA). The measurements were performed using all 12 surface ECG leads and the endocardial channel recorded simultaneously, digital callipers and fast sweep speed (100 mm/s). At least three QRS complexes were measured, and the values were averaged.

### 2.3. Definition of LBB capture and LVS capture

LBB capture was defined if unipolar paced QRS morphology in lead V1 showed QR/rSR′ pattern and at least one of the following:

(a) Transition from ns-LBBP to s-LBBP during differential output pacing characterized by distinct isoelectric interval before the local EGM and the appearance of M/rsR′ pattern and wide R′ with a notch in lead V1, S wave in V5/V6, with constant V6-RWPT.
(b) Transition from ns-LBBP to LVS capture defined by an abrupt prolongation of V6-RWPT ≥10 ms during differential output pacing (6).
(c) When the above QRS transition criteria were not achieved, we used the combined ECG-based criterion of either V6-RWPT < 75 ms or V6-V1 interpeak interval ≥ 33 ms to discriminate ns-LBBP from LVSP (9).

**Figure 1:**
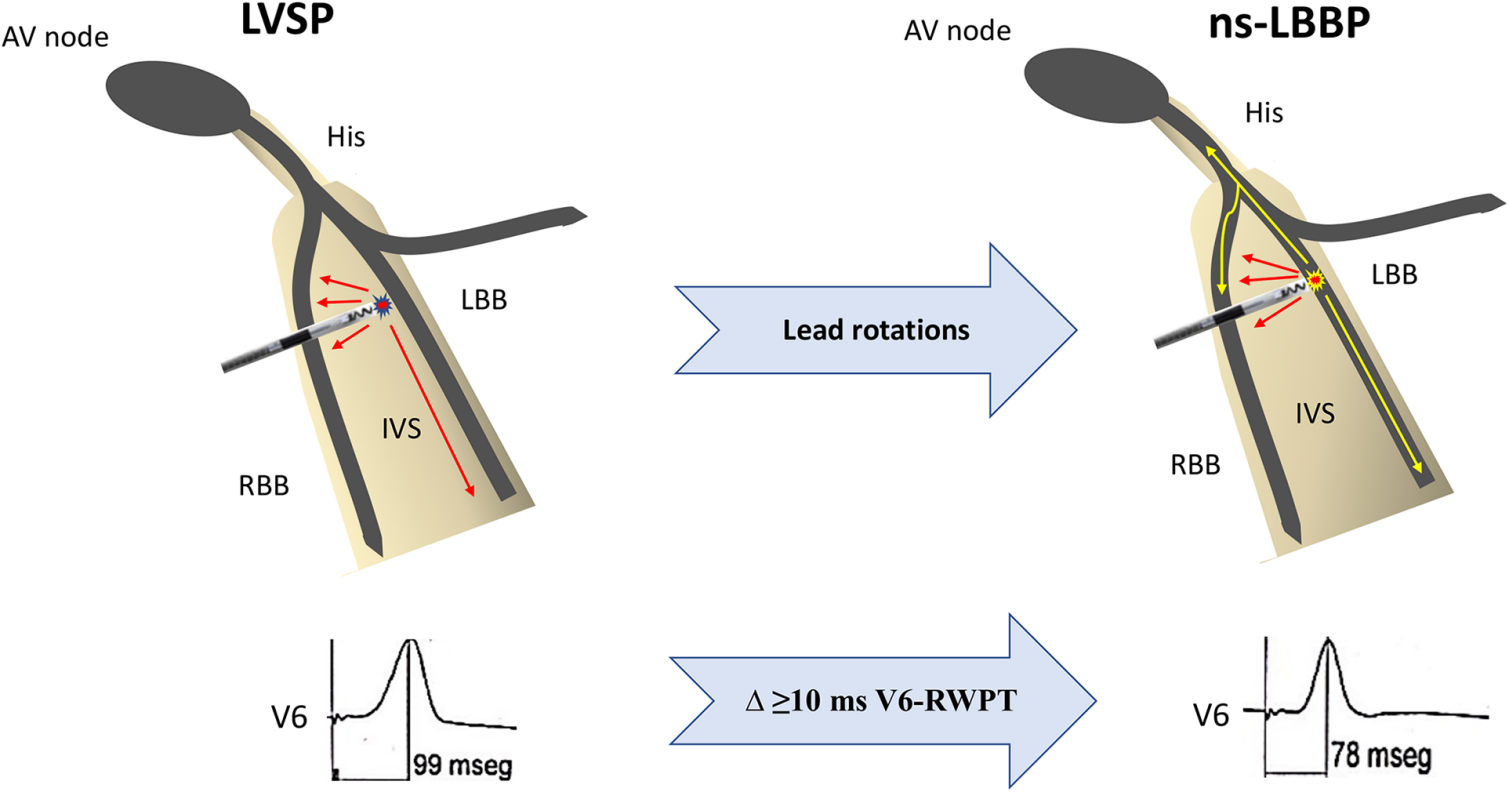
Schematic representation of QRS transition during lead screwing-in. Left ventricular septal morphology is accomplished as the lead reach the LBB area but not the left bundle branch. With additional rotations, the lead finally reaches the conduction system and therefore a ns-LBBP morphology appears. Shortening of at least 10 ms in paced V6-RWPT suggests LBB capture. AV, atrioventricular; IVS, interventricular septum; LBB, left bundle branch; LVSP, left ventricular septal pacing; ns-LBBP, non-selective left bundle branch pacing; RBB, right bundle branch; RWPT, R wave peak time.

QRS transition during lead screwing-in was defined as shortening of paced V6-RWPT by ≥10 ms from LVSP morphology to ns-LBBP morphology, obtained with interrupted pacemapping, during mid to deep septal lead progression at the same target area, between two consecutive pacing manoeuvres, deep and deeper, with a QR/rSR′ pattern in V1 in both (Figure 1). It was mandatory that paced V6-RWPT remained short and constant in the deeper LBBP position, at high and low output pacing.

LVSP was defined when the paced QRS morphology at deep septal location showed a QR or QS pattern in lead V1, R wave without any notch in lead V6, and none of the above LBBP criteria were met.

### 2.4. Measurements and LBBP morphologies

In each patient, every available paced QRS type (s-LBB, ns-LBB and LVSP) and native QRS were measured. The following QRS characteristics were obtained:

(1) Native QRS duration and paced QRS duration measured from the pacing stimulus and from the earlier onset to the latest offset of the QRS in any of the 12 ECG leads recorded simultaneously.
(2) RWPT, measured from the beginning of the pacing spike to the peak of R wave in lead V6 and aVL.
(3) V6-V1 interpeak interval, measured from the R-wave peak in lead V6 to the R-wave peak in lead V1 during simultaneous recording.
(4) The different morphologies of LBBP attending to the site of pacing at the LBB system were analysed, to consider whether they corresponded to left bundle trunk pacing or left fascicle pacing. The different types of pacing sites were considered as following (16, 17):
  • Left bundle trunk pacing (LBTP): Potential-ventricular EGM (vEGM) interval of 25–35 ms and QRS axis similar to sinus rhythm.
  • Left anterior fascicular pacing (LAFP): dominant S wave in leads I and aVL, dominant R wave in leads II, III, and aVF, right-axis deviation, with potential-vEGM interval <25 ms or without potential.
  • Left septal fascicular pacing (LSFP): potential-vEGM interval <25 ms or without potential and QRS axis similar to sinus rhythm or inferior axis with negative component in lead III.
  • Left posterior fascicular pacing (LPFP): dominant R wave in leads I and aVL, dominant S wave in leads II, III, and aVF, left-axis deviation, with potential-vEGM interval <25 ms or without potential.

### 2.5. Statistical analysis

Continuous variables were expressed as mean ± standard deviation (SD) and categorical data as numbers or percentages. Continuous variables were compared using the Student *t-*test or the Mann–Whitney *U*-test, as appropriate. Categorical variables were compared using *χ*^2^, or the Fisher exact test when the conditions required for the former test were not met. For paired comparisons, Student *t-*test was used for Gaussian variables, Wilcoxon non-parametric test for non-Gaussian variables and McNemar-Broker test for categorical variables. The predictive value of the optimal LBBP score cutoff value (≥3 points) (11) for the discrimination of ns-LBBP from LVSP was assessed using standard measures [sensitivity (SN), specificity (SP)] among the study groups of patients according to the type of QRS transition (dynamic manoeuvres vs. lead screwing-in). The data managements and analyses were performed with SPSS, version 20.0 (IBM corporation, Chicago, Illinois). Significance was defined as *p* < 0.05.

## 3. Results

**Figure 2:**
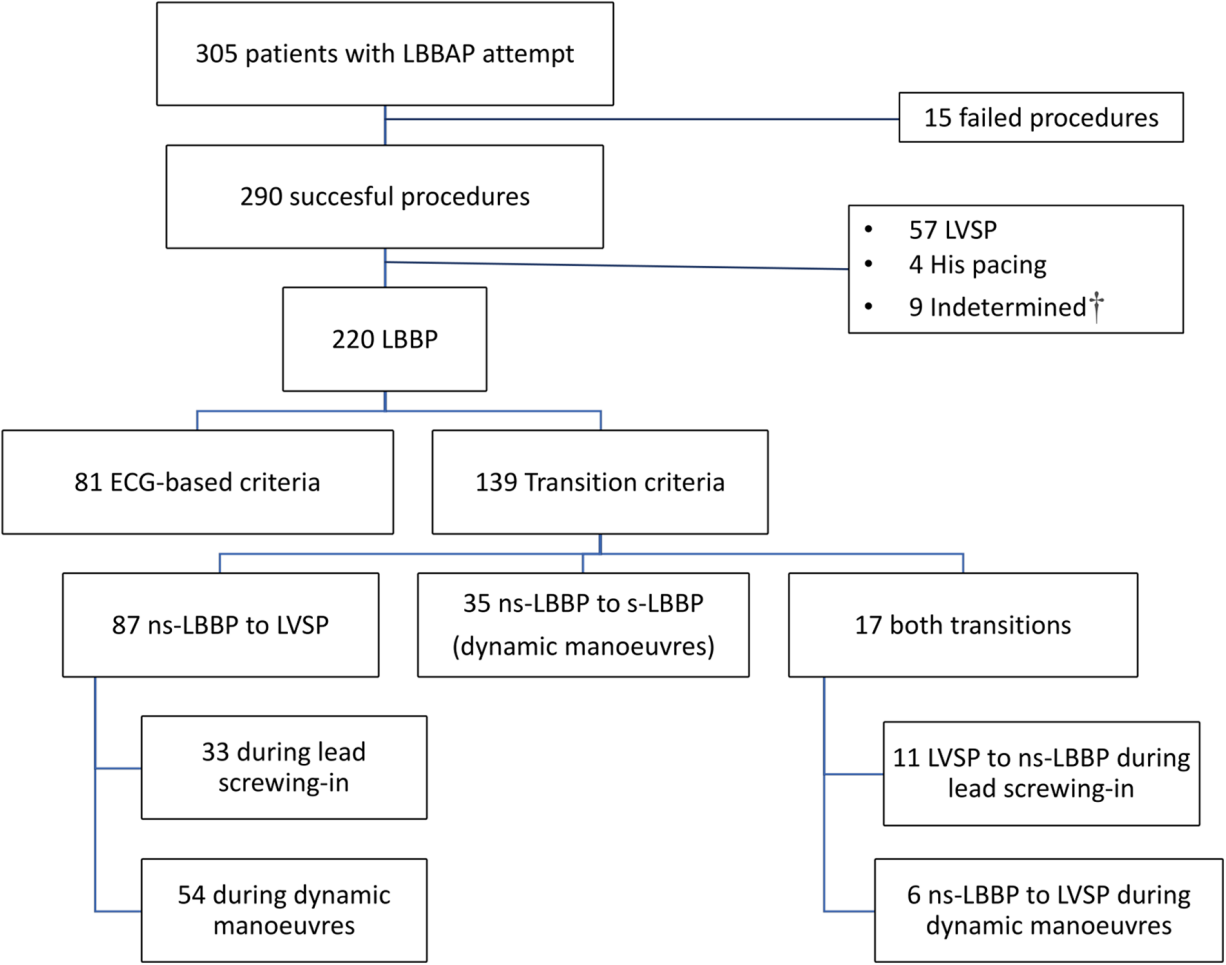
Study population enrollment flowchart. LBBP, left bundle branch pacing; LBBAP, left bundle branch area pacing LVSP, left ventricular septal pacing; ns-LBBP, non-selective left bundle branch pacing; s-LBBP, selective left bundle branch pacing. †Indetermined: patients with LBBAP morphology but without definitive characterization due to overlap criteria for LVSP and ns-LBBP capture.

A total of 305 patients with intended LBBAP were screened. Figure 2 shows the study population flowchart. Successful LBBAP was accomplished in 95.1% of procedures with direct LBB capture demonstrated in 220 patients (75.9%) and LVSP capture achieved in 18.7% of patients. Transition in QRS morphology occurred in 139 out of 290 successful procedures (47.9%). In 17 patients both QRS transitions (from ns-LBBP to s-LBBP and from LVSP to ns-LBBP) were observed. The final study population consisted of 104 patients with transition from left ventricular septal capture to ns-LBBP: forty-four patients with transition during lead screwing-in were compared to 60 cases with a demonstrated transition criterion during pacing manoeuvres.

### 3.1. Baseline characteristics

Table 1 and Supplementary Table S1 show the baseline characteristics of the whole study population and the study groups according to the moment of QRS transition. Majority of patients received a device due to bradycardia pacing indications (93.3%) and only 12.7% of patients presented with left ventricular ejection fraction below 40%. Non-diseased LBB [narrow QRS complex or isolated right bundle branch block (RBBB)] was present in 71.2% of patients and only 9.6% (*n* = 10) of patients showed complete LBB block (LBBB). There were no significant differences in baseline characteristics among study groups, especially regarding left chambers size and function, interventricular septum (IVS) thickness and baseline QRS parameters (QRS width and distributions of bundle branch block). A trend towards a lower body mass index was found among patients in which QRS transition was observed during lead screwing-in.

**Table 1:** Baseline characteristics according to the type of transition from ns-LBBP to LVSP.

	Dynamic manoeuvres (*n* = 60)	Lead screwing-in (*n* = 44)	*p* value
Clinical variables			
Age (years)	79.1 ± 10.3	76.6 ± 9.6	0.216
BMI	29.7 ± 6.1	27.6 ± 4.3	0.051
Male	27 (45)	25 (56.8)	0.234
Hypertension	53 (88.3)	34 (77.3)	0.132
Diabetes mellitus	19 (31.7)	13 (29.5)	0.817
AF	29 (48.3)	21 (48.3)	0.951
CKD^a^	11 (18.6)	7 (15.9)	0.718
Coronary heart disease	10 (16.7)	4 (9.1)	0.263
COPD	7 (11.7)	3 (6.8)	0.407
Previous heart failure	14 (23.3)	11 (25)	0.844
Pacing indication			0.424
AV block	23 (38.3)	13 (30.2)	
Slow AF/bradycardia-tachycardia syndrome	25 (41.6)	16 (37.2)	
Sinus node disease	7 (11.7)	7 (15.9)	
CRT	3 (5)	4 (9.3)	
Bifascicular block + syncope/alternant BBB	2 (3.3)	4 (9.3)	
Echocardiographic parameters			
LVEF (%)	57.3 ± 11.1	57.6 ± 11.4	0.898
LVEF < 40%	7 (11.9)	6 (14)	0.755
LVEDD (mm)	46.1 ± 6.6	47.4 ± 5.8	0.360
IVS thickness (mm)	12.3 ± 3.2	11.8 ± 2.8	0.438
Left atrial volume (ml/m^2^)	45.7 ± 18.0	50.5 ± 20.2	0.235
Baseline ECG characteristics			
PR interval	184.9 ± 54.5	190.2 ± 74.8	0.792
Native QRS width (ms)	113.4 ± 29.7	119.3 ± 34.8	0.348
QTc interval	436.3 ± 37.1	433.9 ± 38.3	0.809
Wide QRS complex (>120 ms)	23 (38.3)	20 (45.5)	0.466
**Baseline ECG morphology** ^b^			0.687
Isolated RBBB	7 (25.9)	6 (28.6)	
RBBB + LFB	8 (29.6)	7 (33.4)	
LBBB	6 (22.2)	4 (19)	
NIVCD	1 (3.7)	0 (0)	
Asystole/PM dependent	1 (3.7)	3 (14.3)	

Values are mean ± standard deviation (SD) and *n* (%). AF, atrial fibrillation; AV, atrioventricular; BBB, bundle branch block; BMI, body mass index; CKD, Chronic kidney disease; COPD, Chronic obstructive pulmonary disease; CRT, cardiac resynchronization therapy; IVS, Interventricular septum; LBBB, left bundle branch block; LFB, left fascicular block; LVEDD, Left ventricular end-diastolic diameter; LVEF, left ventricular ejection fraction; LVSP, left ventricular septal pacing; ns-LBBP, non selective-left bundle branch pacing; PM, pacemaker; RBBB, right bundle branch block.

^a^ Glomerular Filtration rate <60 ml/min/1.73 m^2^.

^b^ Percentages related to wide QRS complex patients.

### 3.2. Procedural and electrical parameters

Table 2 and Supplementary Table S2 display data related to the implantation procedures depending on the type of QRS transition observed. The average time spent for the LBBP lead implantation (from sheath introduction to sheath removal) was 19.1 ± 18.5 min. There were no differences between native QRS duration and final paced QRS duration (ns-LBBP morphology) among study groups (119.3 ± 34.8 vs. 116.6 ± 17.7 ms; *p* = 0.433 for cases with transition during lead screwing-in; and 113.4 ± 29.7 vs. 111.8 ± 16.2 ms; *p* = 0.614 for cases with transition during dynamic manoeuvres). Left conduction system potential was found in 58 cases (56.9%) and the most frequent capture type was left septal fascicular pacing (*n* = 39). Procedural characteristics did not differ among study groups, although there were significantly higher rates of left fascicular pacing (vs. left bundle trunk pacing; *p* = 0.032) among patients in which QRS transition appeared during lead screwing-in.

**Table 2:** Comparison of procedural characteristics according to the type of transition from ns-LBBP to LVSP.

	Dynamic manoeuvres (*n* = 60)	Lead screwing-in (*n* = 44)	*p* value
LBBAP lead placement			
Fluoroscopy (min)	8.3 ± 9.1	10.4 ± 11.7	0.310
Time (min)	18.0 ± 17.9	20.4 ± 19.5	0.546
Paced QRS morphology (ns-LBBP)			
QRS duration (from onset) (ms)	111.8 ± 16.2	116.6 ± 17.7	0.165
QRS duration (from stimulus) (ms)	144.7 ± 19.2	149.5 ± 22.4	0.249
LB potential	35 (60.3)	22 (51.2)	0.357
LB potential to QRS onset (ms)	21.7 ± 6.8	20.1 ± 6.5	0.388
Type of LBB capture			0.032
LBB trunk	12 (20.7)	2 (5.1)	
Fascicular capture	46 (79.3%)	37 (94.9%)	
Left anterior fascicle	4 (8.7)	5 (13.5)	
Left posterior fascicle	16 (34.8)	17 (45.9)	
Left septal fascicle	26 (56.5)	15 (40.6)	
Electrical parameters (acute setting)			
R wave sensing (mV)	9.2 ± 4.2	10.0 ± 4.4	0.386
Impedance (Ohm)	1011.8 ± 232.5	940.9 ± 191.7	0.102
Threshold (Volts) (×0.4 ms)	1.1 ± 0.7	0.8 ± 0.4	0.042
**Type of device implanted**			0.743
SR	19 (31.7)	11 (25.6)	
DR	38 (63.3)	29 (65.9)	
CRT-P	1 (1.6)	2 (4.6)	
CRT-ICD	2 (3.3)	2 (4.6)	

Values are mean ± standard deviation (SD) and *n* (%).

CRT-P, cardiac resynchronization therapy-pacemaker; CRT-ICD, cardiac resynchronization therapy-implantable cardioverter defibrillator; LB, left bundle; LBB, left bundle branch; LVSP, left ventricular septal pacing; ns-LBBP, nonselective left bundle branch pacing; RWPT, R wave peak time.

### 3.3. Comparison of pattern and ECG-based criteria between LVSP and ns-LBBP morphologies

Overall, mean change in paced V6-RWPT from LVSP to ns-LBB capture was 17.1 ± 6.1 ms. Average shortening in paced V6-RWPT was similar among study groups (17.3 ± 6.8 ms in patients with QRS transition during dynamic manoeuvres vs. 18.8 ± 4.9 ms in patients with QRS transition during lead screwing-in; *p* = 0.719). Table 3 shows the comparison of three ECG-based criteria among ns-LBBP and LVSP morphologies according to the moment of QRS transition. LVSP morphologies showed significant longer paced V6-RWPT and paced aVL-RWPT, and shorter V6-V1 interpeak interval (*p* < 0.001 for the three comparisons) compared to ns-LBBP morphologies among the two QRS transition groups.

**Table 3:** ECG-based criteria for the differentiation of ns-LBBP from LVSP according to the type of transition.

	ns-LBBP morphologies			LVSP morphologies		
	Dynamic manoeuvres (*n* = 60)	Lead screwing-in (*n* = 44)	*p* value	Dynamic manoeuvres (*n* = 60)	Lead screwing-in (*n* = 44)	*p* value
qR/rSr′ pattern (lead V1)	59 (98.3)	44 (100)	0.577	57 (95)	38 (86.4)	0.117
Paced V6-RWPT (ms)	78.1 ± 8.9	75.0 ± 9.6	0.067	95.4 ± 12.3	91.9 ± 9.2	0.159
V6-V1 interpeak (ms)	39.6 ± 11.3	43.0 ± 12.3	0.125	28.3 ± 9.9	25.1 ± 10.8	0.109
Paced aVL-RWPT (ms)	78.7 ± 12.1	79.4 ± 16.5	0.977	93.3 ± 15.5	92.4 ± 17.8	0.699
LBBP score (mean)	4.3 ± 1.8	5.1 ± 2.2	0.094	0.8 ± 1.2	0.7 ± 1.2	0.741

Values are mean ± standard deviation (SD).

LBBP, left bundle branch pacing; LVSP, left ventricular septal pacing; ns-LBBP, nonselective left bundle branch pacing; RWPT, R wave peak time.

No significant differences were found in ns-LBBP morphologies between QRS transition study groups, but a trend towards shorter paced V6-RWPT was found in ns-LBBP morphologies obtained during lead penetration (Table 3).

Besides, there were not significant differences among the ns-LBBP morphologies in the lead screwing transition group related to the concomitant presence of non-selective to selective LBBP transition (Supplementary Table S3).

### 3.4. Completion of 100% specific ECG-based criteria cut-off values

Sixty one out of 104 ns-LBBP morphologies (58.7%) met the 100% specific cut-off values of V6-RWTP < 75 ms or V6-V1 interpeak interval > 44 ms, which was more frequently achieved in patients with transition during lead screwing-in (*n* = 31) compared to patients with transition during dynamic manoeuvres (*n* = 30) (70.5% vs. 50%, respectively; *p* = 0.036). On the contrary, only 4.8% of LVSP morphologies (*n* = 5) met the 100% specific criterion of V6-RWTP < 75 ms or V6-V1 interpeak interval > 44 ms, without differences among patients with transition during lead screwing-in (*n* = 2) and patients with transition during dynamic manoeuvres (*n* = 3) (4.5% vs. 5%, respectively; *p* = 0.990).

Diagnostic performance for QRS transition during lead screwing-in was assessed in the population with defined LBB capture (*n* = 220), based on the completion of the combined criterion of V6-RWPT < 75 ms or V6-V1 interpeak interval > 44 ms. A total of 157 patients accomplished one of these cut-off values. Therefore, estimated SN for transition during lead screwing-in was 19.8% and estimated SP was 82.5% (52 out of 63 patients who did not accomplish any of the 100% specific cut-off values, had no transition during lead screwing-in).

### 3.5. Performance of LBBP score among different types of QRS transition

As expected, mean LBBP score was significantly higher in ns-LBBP morphologies than in LVSP morphologies among both QRS transition groups (*p* < 0.001 for both comparisons). Average LBBP score was similar in LVSP morphologies obtained during lead screwing-in and during dynamic manoeuvers. However, a trend towards higher LBBP score was found in ns-LBBP morphologies in patients in which QRS transition was obtained during lead penetration compared to patients in which QRS transition was demonstrated during dynamic manoeuvers (5.1 ± 2.2 vs. 4.3 ± 1.8, respectively; *p* = 0.094).

Performance of the optimal LBBP score cut-off value (≥3 points) to discriminate LVS capture from ns-LBB capture showed SN of 89.7% and a SP of 94.1% among cases with QRS transition during lead screwing-in and SN of 78% and SP of 91.5% among cases with QRS transition demonstrated during dynamic manoeuvers. In patients with non-diseased LBB (narrow QRS complex or isolated RBBB patients) the optimal LBBP score cut-off point of ≥3, displayed SN of 84.6% and SP of 95.7% among cases with QRS transition during lead screwing-in and SN of 80.6% and SP of 97% among cases with QRS transition demonstrated during dynamic manoeuvers.

### 3.6. Acute complications

There was one episode of ventricular fibrillation during lead penetration, solved with defibrillation, without further consequences. Complications rate at 30-days was 15.4% (*n* = 16), mainly driven by septal perforation (*n* = 8) followed by acute pacing threshold >2 V × 0.4 ms (*n* = 5), pneumothorax (*n* = 2), intraprocedural lead dislodgement (*n* = 1) and acute chest pain (*n* = 1). No stroke or other thromboembolic complications were observed in cases of perforations of the lead into the LV cavity. Lead repositioning was feasible in cases of septal perforation or lead dislodgement without further complications. No deaths, infection or cardiac perforation were observed at 30-days after the implant.

No significant differences in complications rate were observed between QRS transition groups (18.2% vs. 13.3% for QRS transition during lead screwing-in and dynamic manoeuvers, respectively; *p* = 0.498) but septal perforation was significantly more frequent in cases of QRS transition observed during lead screwing-in compared to cases in which QRS transition was demonstrated during dynamic manoeuvers: 15.9% vs. 1.7%, respectively (*p* = 0.01).

## 4. Discussion

### 4.1. Paced QRS morphologies during LBBAP procedure and demonstration of QRS transition

Loss of myocardial capture during LBBAP, also called “selective response” (transition from ns-LBBP to s-LBBP), can be demonstrated during dynamic manoeuvers by several changes in paced QRS morphology with constant V6-RWPT (7, 8). However discerning pure myocardial pacing from non-selective conduction system pacing requires careful look at the different paced QRS morphologies. Transition from ns-LBBP to LVSP (“myocardial response”) was first described by Jastrzębski et al. in a very elegant work by performing both deep septal programmed stimulation and differential output pacing (7). Indeed, myocardial response was more frequently observed by dynamic manoeuvers than selective response. An invasive validation of these QRS transition criteria was done by Wu et al. (6), showing 100% SP. Therefore, transition from ns-LBBP to LVS capture was universally accepted if an abrupt prolongation of V6-RWPT ≥ 10 ms during differential output pacing occurred. Although QRS transition criteria have been adopted as the non-invasive gold-standard criteria for LBB capture they are difficult to demonstrate in clinical practice. Reported rates of QRS transition criteria during dynamic manoeuvers among the different LBBAP series ranges from 30% to 60% (7–9, 11, 18) and the recent multicenter European experience showed transition in only 23.6% of procedures (16). That is the reason why several ECG-based criteria have been proposed to help physicians in confirming LBB capture (9–11).

Since the first descriptions of the LBBAP technique, pacemapping during lead screwing-in is the most recommended method to monitor paced QRS changes as the lead is progressively deeper deployed into the interventricular septum (19). Pacemapping can be performed with or without interruption of pacing, but it is usually needed to detach the connector-pin to provide rapid rotations in order to avoid lead damage (14). If continuous pacemapping is performed during lead rotations (uninterrupted pacemapping) a dynamic QRS transition can be observed from wide QRS complexes with LBBB morphology and a notched nadir in lead V1 to narrow QRS complexes with RBBB pattern in lead V1, that accomplish LBB capture criteria (7, 20). Paced morphologies compatible with LVSP usually appear several rotations before the final LBB capture morphology emerges.

**Figure 3:**
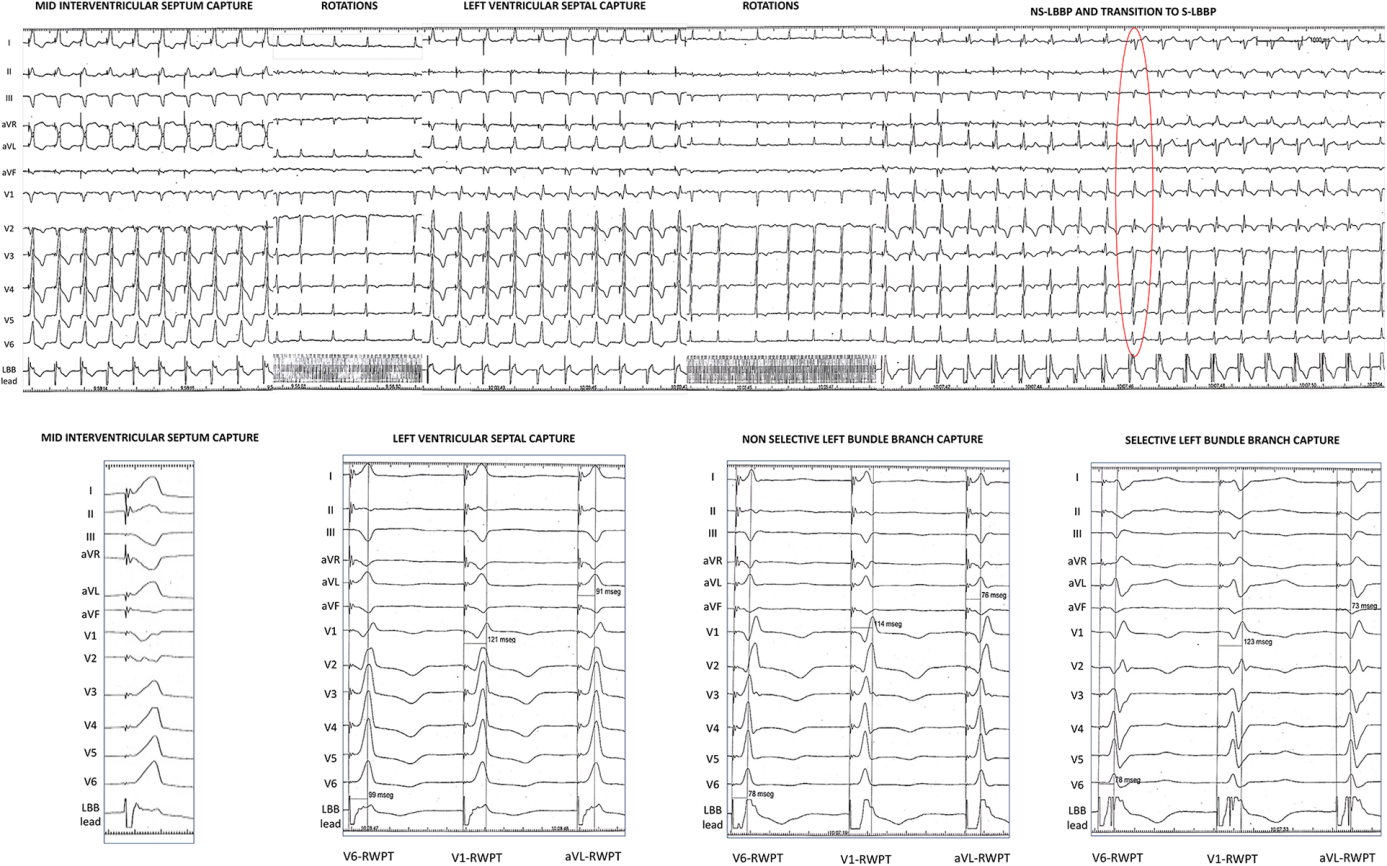
Example of QRS transition during lead screwing-in. Top panel: collage of paced QRS morphologies obtained as the lead was progressively deeper deployed into the interventricular septum with interruption of pacing (marked as lead rotations). Red circle shows the first paced beat with s-LBBP. Bottom part: zoom on the different paced QRS morphologies found during lead screwing-in with three ECG-based criteria annotated. Left ventricular septal capture shows a V6-V1 interpeak interval of 22 ms and a LBBP score punctuation of 0; non-selective left bundle branch pacing capture shows a V6-V1 interpeak interval of 36 ms and a LBBP score punctuation of 5; and selective left bundle branch capture shows a V6-V1 interpeak interval of 45 ms and a LBBP score punctuation of 8. ns-LBBP, non-selective left bundle branch pacing; RWPT, R wave peak time; s-LBBP, selective left bundle branch pacing.

The same phenomenon can be also seen with interrupted lead screwing-in pacemapping or even with the occurrence of induced ectopic beats (fixation beats) (21). As the lead progresses deep into the septum, LVSP morphologies, with relative narrow QRS complex but delayed V6-RWPT, can be observed once the lead reaches the area of the LBB but not the conduction system. After several rotations the lead finally comes in contact with the LBB and ns-LBBP morphology occurs (Figures 1, 3). At this point, dynamic manoeuvers might be able to demonstrate transition to LVSP or even to s-LBBP, but as mentioned before, similar pacing thresholds between the LBB and the adjacent myocardium limit this response frequently.

### 4.2. QRS transition during lead screwing-in

To the best of our knowledge, QRS transition during lead screwing-in has never been formally described, although it was used in the LBBP-RESYNC trial (22). To define QRS transition during lead screwing-in we took into consideration the same cut-off point (at least 10 ms of change in paced V6-RWPT) used to define transition during dynamic manoeuvers, which has a reported specificity of 100%. ECG-based criteria were used to confirm final LBB captures and to discriminate them from LVSP captures. There were no significant differences between ns-LBBP captures obtained during dynamic manoeuvers and those obtained during lead screwing-in, nor even between LVSP captures obtained under both methods. Indeed, more ns-LBBP morphologies obtained during lead screwing-in met the 100% specific criterion of V6-RWPT < 75 or V6-V1 interpeak interval > 44 ms compared to those ns-LBBP morphologies obtained during threshold test, which supports the plausibility of this tool. We believe that the occurrence of transition from LVSP to ns-LBBP during lead penetration into the IVS can be a marker of LBB capture, especially useful in cases in which differential output pacing fail to demonstrate QRS transition at the final lead position but paced QRS morphology suggest LBB capture based on physiological ECG criteria. Besides, it might help to increase sensitivity of QRS transition criteria, as prevalence of QRS transition increased from 32.7% to 47.9% after considering transition during lead screwing-in.

Although SN and SP could not be accurately assessed, estimated SN using the surrogate of the 100% specific combined criterion (V6-RWPT < 75 ms or V6-V1 interpeak interval > 44 ms) increased from 19.1% (for transition during pacing) to 38.9% when transition during lead screwing-in was also considered.

It is remarkable that left ventricular chambers size and IVS thickness was similar among patients with both types of QRS transition (dynamic manoeuvers and lead screwing-in), which does not allow us to predict which patients can show one QRS transition or the other before the lead deployment. This also applies to QRS width and morphology as transition during lead screwing-in was as frequent as transition during differential output pacing among wide and narrow QRS patients.

Interestingly, QRS transition during lead screwing-in seemed to be more feasible in cases in which left bundle fascicles are targeted to deliver the lead. This is important, as the most frequent type of capture reported in clinical practice is left bundle fascicular capture (16) So, the chance to obtain this type of transition appears to be less frequent when we target basal LBB trunk.

It needs to be emphasized that, although overall complications rate was not different among both types of transition, LV septal perforation was more frequent in the group with lead screwing-in transition. This might be related to the lack of demonstration of QRS transition during dynamic manoeuvers at deep IVS location in that group, and it highlights the importance of combining different methods to confirm LBB capture. The application of ECG-based criteria to deep IVS morphologies obtained during interrupted pacemapping are very useful to stop rotations and to avoid lead protrusion into the LV cavity.

### 4.3. Limitations

This study was conducted in a single center and the definition of QRS transition during lead screwing-in was described in a relatively small cohort, which requires further external validation. We did not perform an invasive validation by placing a multielectrode catheter along the IVS and another one in the His area to confirm the presence or absence of retrograde His potential or distal anterograde left conduction system potential during interrupted lead screwing-in pacemapping. It is extremely complicated to carry out this ideal method for logistic and ethical considerations. Although we used ECG-based criteria with reported 100% SP to support our results, we must admit that this cannot be considered the gold standard method, because these criteria have never been invasively validated. Finally, we did not measure the depth that the lead penetrated into the IVS or compare among study groups. As mentioned before, we do not routinely use contrast injection through the delivery sheath.

## 5. Conclusions

QRS transition from left ventricular septal capture to LBB capture can be observed as the lead penetrates deep into the IVS by interrupted pacemapping during LBBAP procedure. Shortening of at least 10 ms in paced V6-RWPT of QRS complexes resulting from interrupted lead screwing-in pacemapping seems to be practical to confirm the capture of the conduction system and might serve as marker of LBB capture.

## Data Availability

The raw data supporting the conclusions of this article will be made available by the authors, without undue reservation.

## References

[1] Sharma PS Patel NR Ravi V Zalavadia DV Dommaraju S Garg V Clinical outcomes of left bundle branch area pacing compared to right ventricular pacing: results from the geisinger-rush conduction system pacing registry. Heart Rhythm. (2022) 19(1):3–11. 10.1016/j.hrthm.2021.08.033 34481985

[2] Heckman LIB Luermans JGLM Curila K Van Stipdonk AMW Westra S Smisek R Comparing ventricular synchrony in left bundle branch and left ventricular septal pacing in pacemaker patients. J Clin Med. (2021) 10(4):822. 33671420 10.3390/jcm10040822 PMC7923157

[3] Zhang W Chen L Zhou X Huang J Zhu S Shen E Resynchronization effects and clinical outcomes during left bundle branch area pacing with and without conduction system capture. Clin Cardiol. (2023) 46(3):287–95. 36597668 10.1002/clc.23969 PMC10018083

[4] Jastrzębski M Moskal P Huybrechts W Curila K Sreekumar P Rademakers LM Left bundle branch-optimized cardiac resynchronization therapy (LOT-CRT): results from an international LBBAP collaborative study group. Heart Rhythm. (2022) 19(1):13–21. 10.1016/j.hrthm.2021.07.057 34339851

[5] Burri H Jastrzebski M Vijayaraman P. Electrocardiographic analysis for his bundle pacing at implantation and follow-up. JACC Clin Electrophysiol. (2020) 6(7):883–900. 10.1016/j.jacep.2020.03.005 32703577

[6] Wu S Chen X Wang S Xu L Xiao F Huang Z Evaluation of the criteria to distinguish left bundle branch pacing from left ventricular septal pacing. JACC Clin Electrophysiol. (2021) 7(9):1166–77. 10.1016/j.jacep.2021.02.018 33933414

[7] Jastrzębski M Moskal P Bednarek A Kiełbasa G Kusiak A Sondej T Programmed deep septal stimulation: a novel maneuver for the diagnosis of left bundle branch capture during permanent pacing. J Cardiovasc Electrophysiol. (2020) 31(2):485–93. 10.1111/jce.14352 31930753

[8] Vijayaraman P Subzposh FA Naperkowski A Panikkath R John K Mascarenhas V Prospective evaluation of feasibility and electrophysiologic and echocardiographic characteristics of left bundle branch area pacing. Heart Rhythm. (2019) 16(12):1774–82. 10.1016/j.hrthm.2019.05.011 31136869

[9] Jastrzębski M Burri H Kiełbasa G Curila K Moskal P Bednarek A The V6-V1 interpeak interval: a novel criterion for the diagnosis of left bundle branch capture. Europace. (2022) 24(1):40–7. 10.1093/europace/euab164 34255038 PMC8742628

[10] Jastrzębski M Kiełbasa G Curila K Moskal P Bednarek A Rajzer M Physiology-based electrocardiographic criteria for left bundle branch capture. Heart Rhythm. (2021) 18(6):935–43. 10.1016/j.hrthm.2021.02.021 33677102

[11] Briongos-Figuero S Estévez-Paniagua Á Sánchez-Hernández A Muñoz-Aguilera R. Combination of current and new electrocardiographic-based criteria: a novel score for the discrimination of left bundle branch capture. Europace. (2023) 25(3):1051–9. 10.1093/europace/euac276 36691717 PMC10062292

[12] Burri H Jastrzebski M Cano Ó Čurila K de Pooter J Huang W EHRA clinical consensus statement on conduction system pacing implantation: endorsed by the Asia pacific heart rhythm society (APHRS), Canadian heart rhythm society (CHRS), and Latin American heart rhythm society (LAHRS). eur eur pacing, arrhythmias, card electrophysiol J work groups card pacing, arrhythmias. Card Cell Electrophysiol Eur Soc Cardiol. (2023) 25(4):1208–36. 10.1093/europace/euad043 PMC10105878 37061848

[13] Huang W Chen X Su L Wu S Xia X Vijayaraman P. A beginner’s guide to permanent left bundle branch pacing. Heart Rhythm. (2019) 16(12):1791–6. 10.1016/j.hrthm.2019.06.016 31233818

[14] Ponnusamy SS Arora V Namboodiri N Kumar V Kapoor A Vijayaraman P. Left bundle branch pacing: a comprehensive review. J Cardiovasc Electrophysiol. (2020) 31(9):2462–73. 10.1111/jce.14681 32681681

[15] Jastrzębski M Moskal P Hołda MK Strona M Bednarek A Kiełbasa G Deep septal deployment of a thin, lumenless pacing lead: a translational cadaver simulation study. Europace. (2020) 22(1):156–61. 31722391 10.1093/europace/euz270

[16] Jastrzębski M Kiełbasa G Cano O Curila K Heckman L De Pooter J Left bundle branch area pacing outcomes: the multicentre European MELOS study. Eur Heart J. (2022) 43(40):4161–73. 35979843 10.1093/eurheartj/ehac445 PMC9584750

[17] Liu X Gu M Niu H-X Chen X Cai C Zhao J A comparison of the electrophysiological and anatomic characteristics of pacing different branches of the left bundle conduction system. Front Cardiovas Med. (2022) 8:781845. 10.3389/fcvm.2021.781845 PMC8766986 35071354

[18] Chen X Qian Z Zou F Wang Y Zhang X Qiu Y Differentiating left bundle branch pacing and left ventricular septal pacing: an algorithm based on intracardiac electrophysiology. J Cardiovasc Electrophysiol. (2022) 33(3):448–57. 10.1111/jce.15350 34978368

[19] Huang W Su L Wu S Xu L Xiao F Zhou X A novel pacing strategy with low and stable output: pacing the left bundle branch immediately beyond the conduction block. Can J Cardiol. (2017) 33(12):1736.e1–e3. 10.1016/j.cjca.2017.09.013 29173611

[20] Jastrzębski M Moskal P. Reaching the left bundle branch pacing area within 36 heartbeats. Kardiol Pol. (2021) 79(5):587–8. 34125940 10.33963/KP.15914

[21] Jastrzębski M Kiełbasa G Moskal P Bednarek A Kusiak A Sondej T Fixation beats: a novel marker for reaching the left bundle branch area during deep septal lead implantation. Heart Rhythm. (2021) 18(4):562–9. 10.1016/j.hrthm.2020.12.019 33359876

[22] Wang Y Zhu H Hou X Wang Z Zou F Qian Z Randomized trial of left bundle branch vs biventricular pacing for cardiac resynchronization therapy. J Am Coll Cardiol. (2022) 80(13):1205–16. 10.1016/j.jacc.2022.07.019 36137670

