# Supplementary material for: Redefining QRS transition to confirm left bundle branch capture during left bundle branch area pacing
Source: Front Cardiovasc Med. 2023 Jul 14;10:1217133. doi: 10.3389/fcvm.2023.1217133 (PMC10375013; doi:10.3389/fcvm.2023.1217133)
Supplement: Supplementary file 1 [file Table1.docx]

**Supplementary table 1. Baseline characteristics.**

|  | | Study population (n=104) |
| --- | --- | --- |
| **Clinical variables** |  | |
| - Age (years) | 78.0±10.0 | |
| - BMI | 28.9±5.4 | |
| - Male | 52 (50) | |
| - Hypertension | 87 (83.7) | |
| - Diabetes mellitus | 32 (30.8) | |
| - AF | 50 (48.1) | |
| - CKD* | 18 (17.5) | |
| - Coronary heart disease | 14 (13.5) | |
| - COPD | 10 (9.6) | |
| - Previous heart failure | 25 (24) | |
| **Medical treatment** |  | |
| - Anticoagulation | 56 (53.8) | |
| - Beta blockers | 33 (31.7) | |
| - ACEI/ARB | 61 (58.7) | |
| - Sacubitril/Valsartan | 7 (6.7) | |
| - Aldosterone antagonist | 18 (17.6) | |
| **Pacing indication** |  | |
| - AV block | 36 (34.6) | |
| - Slow AF/bradycardia-tachycardia syndrome | 41 (39.5) | |
| - Sinus node disease | 14 (13.5) | |
| - CRT | 7 (6.7) | |
| - Bifascicular block+syncope/alternant BBB | 6 (5.8) | |
| **Echocardiographic parameters** |  | |
| - LVEF (%) | 57.4±11.3 | |
| - LVEF < 40% | 13 (12.7) | |
| - LVEDD (mm) | 46.6± 6.3 | |
| - IVS thickness (mm) | 12.1±3.0 | |
| - Left atrial volume (ml/m2) | 47.8±19.0 | |
| **Baseline ECG characteristics** |  | |
| - PR interval | 186.9±61.5 | |
| - Native QRS width (ms) | 115.9±31.9 | |
| - QTc interval | 435.3±37.3 | |
| - Wide QRS complex (>120 ms) | 43 (41.3) | |
| **Baseline ECG morphology **** |  | |
| - Isolated RBBB | 13 (27.1) | |
| - RBBB + LFB | 15 (31.3) | |
| - LBBB | 10 (20.8) | |
| - NIVCD | 1 (2.1) | |
| - Asystole/PM dependent | 4 (8.3) | |

Values are mean ± standard deviation (SD) and n (%).

*Glomerular filtration rate < 60 ml/kg/1.73 m^2^

** Percentages related to wide QRS complex patients

ACEI: angiotensin-converting enzyme inhibitors; AF: atrial fibrillation; ARB: angiotensin receptor blocker; AV: atrioventricular; BBB: bundle branch block; BMI: body mass index; CKD: Chronic kidney disease; COPD: Chronic obstructive pulmonary disease; CRT: cardiac resynchronization therapy; IVS: Interventricular septum; LBBB: left bundle branch block; LFB: left fascicular block; LVEDD: Left ventricular end-diastolic diameter; LVEF: left ventricular ejection fraction; LVSP: left ventricular septal pacing; ns-LBBP: non selective-left bundle branch pacing; PM: pacemaker; RBBB: right bundle branch block

**Table 2. Procedural characteristics**

|  | Study population (n=104) |
| --- | --- |
| LBBAP lead placement |  |
| - Fluoroscopy (min) | 9.1±10.3 |
| - Time (min) | 19.1±18.5 |
| Paced QRS morphology (ns-LBBP) |  |
| - QRS duration (from onset) (ms) | 113.8±16.9 |
| - QRS duration (from stimulus) (ms) | 146.7±20.7 |
| LB potential | 57 (56.4) |
| LB potential to QRS onset (ms) | 21.2±6.7 |
| Type of LBB capture |  |
| - LBB trunk | 14 (14.4) |
| - Left anterior fascicle | 9 (9.3) |
| - Left posterior fascicle | 33 (34) |
| - Left septal fascicle | 41 (42.3) |
| Electrical parameters (acute setting) |  |
| - R wave sensing (mV) | 9.6±4.3 |
| - Impedance (Ohm) | 981.8±218.1 |
| - Threshold (Volts) (x 0.4 ms) | 0.9±0.6 |
| Type of device implanted |  |
| - SR | 30 (28.8) |
| - DR - CRT-P - CRT-ICD | 67 (64.4)  3 (2.9)  4 (3.8) |

Values are mean ± standard deviation (SD) and n (%).

CRT-P: cardiac resynchronization therapy-pacemaker; CRT-ICD: cardiac resynchronization therapy-implantable cardioverter defibrillator; LB: left bundle; LBB: left bundle branch; LVSP: left ventricular septal pacing; ns-LBBP: nonselective left bundle branch pacing; RWPT: R wave peak time.

**Supplementary table 3. ECG-based criteria in the lead screwing transition group, according to the concomitant presence of non-selective to selective LBBP transition.**

**ns-LBBP morphologies LVSP morphologies**

|  | LVSP to ns-LBBP transition only (n=33) | ns-LBBP to  s-LBBP  (n=11) | p value | LVSP to ns-LBBP transition only (n=33) | ns-LBBP to s-LBBP  (n=11) | p value |
| --- | --- | --- | --- | --- | --- | --- |
| Paced V6-RWPT (ms) | 74.1±8.5 | 77.8±12.3 | 0.505 | 90.3±8.1 | 96.5±10.8 | 0.079 |
| V6-V1 interpeak (ms) | 42.3±13.2 | 45.1±9.2 | 0.260 | 24.9±11.7 | 25.4±7.9 | 0.757 |
| Paced aVL-RWPT (ms) | 77.8±16.1 | 83.5±17.2 | 0.382 | 90.4±19.0 | 97.7±11.7 | 0.219 |
| LBBP score (mean) | 4.9±2.2 | 5.3±1.9 | 0.577 | 0.9±1.3 | 0.4±0.8 | 0.246 |

LBBP: left bundle branch pacing; LVSP: left ventricular septal pacing; ns-LBBP: nonselective left bundle branch pacing; RWPT: R wave peak time; s-LBBP: selective left bundle branch pacing
